# Intracortical intraosseous lipoma

**DOI:** 10.1259/bjrcr.20150280

**Published:** 2016-01-19

**Authors:** Michael Khoo, Ian Pressney, Rikin Hargunani, Paul O'Donnell

**Affiliations:** Department of Radiology, Royal National Orthopaedic Hospital, Stanmore, UK

## Abstract

Intraosseous lipomas are very uncommon, benign primary bone lesions with an incidence of <1%. The overwhelming majority occur within the intramedullary canal. We present an uncommon intracortical intraosseous lipoma with 3 T MRI findings to help differentiate this from other differential diagnoses.

## Summary

Intraosseous lipomas are very uncommon, benign primary bone lesions with an incidence of <1%. The overwhelming majority occur within the intramedullary canal. We present an uncommon intracortical intraosseous lipoma with 3 T MRI findings to help differentiate this from other differential diagnoses.

## Clinical presentation and imaging findings

**Figure 1.**
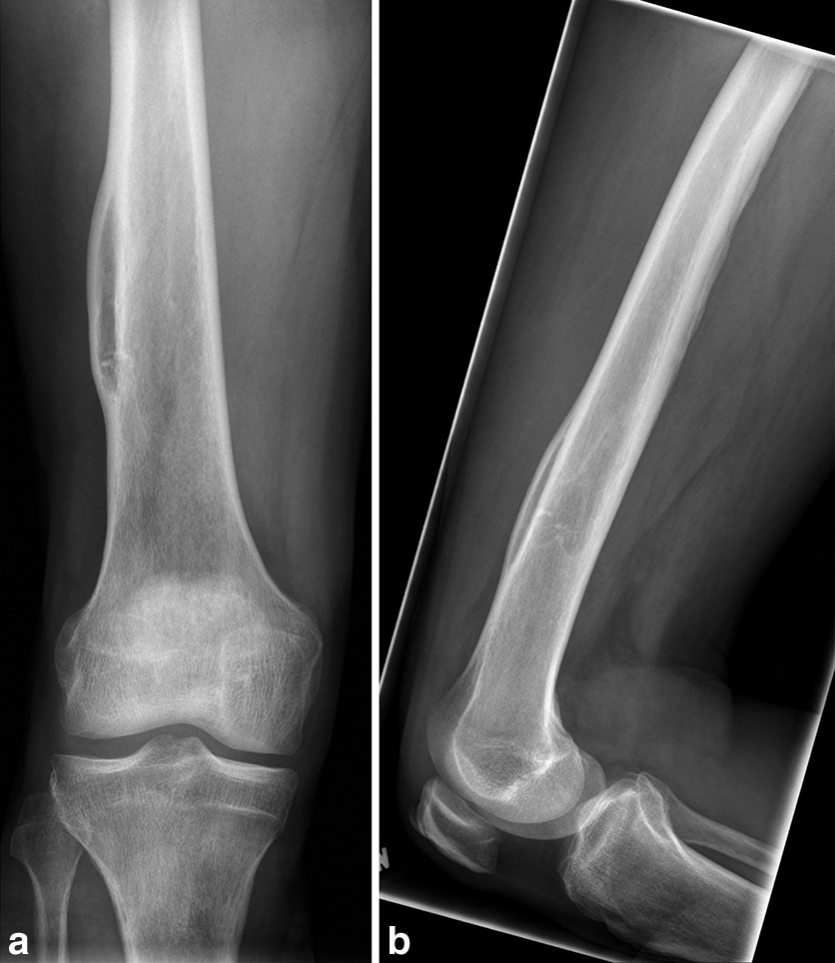
(a) Anterior–posterior and (b) lateral plain radiographs of the distal right femur.

**Figure 2.**
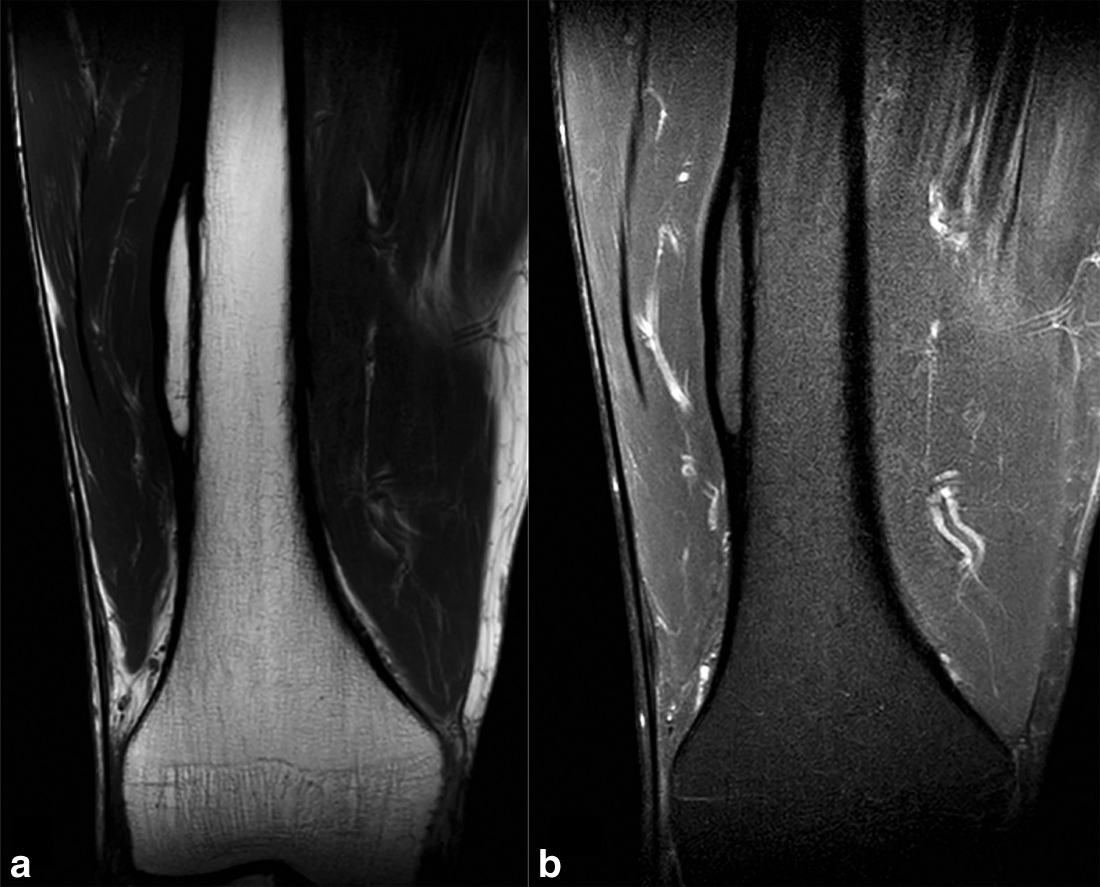
Coronal (a) *T*_1 _weighted and (b) short tau inversion-recovery images of the distal right femur demonstrating an intracortical lesion with completely lipomatous signal characteristics.

**Figure 3.**
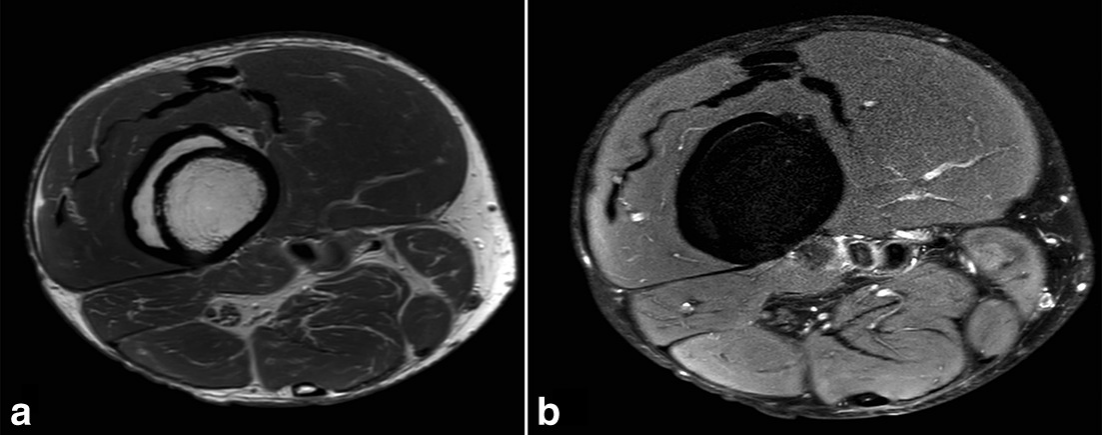
Axial (a) proton density and (b) fat-suppressed proton density images of the distal right femoral lesions demonstrating equal cortical thickness both superficial and deep to the lesion with completely lipomatous intralesional signal characteristics.

A fit and well 57-year-old male with no previous history of recent or past trauma presented with an incidental painless thigh mass and was referred to our tertiary referral sarcoma centre for assessment. Initial plain radiographic assessment (Figure 1) delineated a well-defined, smooth, fusiform intracortical lesion. An MRI examination (Figures 2 and 3) demonstrated a completely enclosed lesion within the femoral cortex returning fat signal characteristics without other tissue, identical to bone marrow within the intramedullary canal. There was no connecting tract between the intracortical lesion and no cystic or fibrous changes, or any evidence of mineralization. There was absence of any aggressive features on imaging.

## Outcome and follow-up

In view of the characteristic findings and the absence of any aggressive features, no needle biopsy was required for this patient, which would have been the usual management pathway for the assessment of an unusual bone lesion. As this was the first presentation, a single follow-up MRI examination locally at 6 months was recommended at the bone tumour multidisciplinary team meeting. Unfortunately, it has not been possible to contact the patient since his imaging review and no follow-up imaging is available at present.

## Discussion

Intraosseous lipomas are very uncommon, benign primary bone lesions with an incidence of <1%^1^ that usually occur within the fourth and fifth decades of life.^2,3^ They can be mildly painful but are predominantly asymptomatic and found incidentally. Intraosseous lipomas often occur within the femur, usually proximally at an intertrochanteric or subtrochanteric position, with the tibia, fibula and calcaneus being the other common locations. The overwhelming majority of cases describe lesions occurring in the intramedullary canal, with only a few reported cases demonstrating an intracortical location,^4–6^ as shown in this case.

Plain radiography demonstrates a well-defined intracortical lesion of low internal density with no evidence of previous injury or aggressive features. Subsequent MRI examination confirms a lesion confined to an intracortical location, with comparable cortical thickness both deep and superficial to the lesion, in contrast to a periosteal lipoma that would present as a mass adjacent to bone. The MRI also confirms the completely homogeneous fat signal of the lesion, which is helpful in differentiating it from intracortical fibrous lesions such as osteofibrous dysplasia that would typically demonstrate more heterogeneous signal from their non-fatty components. The more common intramedullary intraosseous lipoma can demonstrate additional imaging characteristics, including calcification and cystic change,^2,3^ but this has not been widely reported in the intracortical variety.^4–6^ The MRI also demonstrates no continuity of the lesion with the intramedullary canal marrow, helping differentiate it from potential post-traumatic entry of marrow into the cortex and other healed lesions such as a fibrous cortical defect or infection.

In keeping with the few cases reported in the literature, our case did not demonstrate any aggressive features, with absence of non-lipomatous elements, periosteal reaction and associated extraosseous mass. For this patient, recognition of the homogeneous and uniform fat signal characteristics within a painless, non-aggressive lesion to demonstrate an intracortical intraosseous lipoma resulted in the patient avoiding unnecessary needle biopsy or surgical resection and the associated morbidity.

## Learning points

1. Intraosseous lipomas are very uncommon, benign primary bone lesions with an incidence of <1%.
2. The vast majority occur in the intramedullary canal but, as in this case, rarely, it can be present in an intracortical location.
3. Features alluding to an intracortical intraosseous lipoma include:
  - completely lipomatous signal characteristics
  - intracortical location without any communication with normal intramedullary bone marrow
  - comparable cortical thickness, both superficial and deep, around the lesion
  - non-aggressive imaging features.
